# Differential impact of student behaviours on group interaction and collaborative learning: medical students’ and tutors’ perspectives

**DOI:** 10.1186/s12909-016-0730-1

**Published:** 2016-08-22

**Authors:** Maha Iqbal, Gary M. Velan, Anthony J. O’Sullivan, Chinthaka Balasooriya

**Affiliations:** 1 School of Public Health and Community Medicine, UNSW Medicine, UNSW Australia, Sydney, 2052 Australia; 2 Educational Research and Development Unit at the School of Medical Sciences, UNSW Medicine, University of New South Wales, Sydney, Australia; 3 St. George and Sutherland Clinical School and Program Authority, UNSW Medicine, University of New South Wales, Sydney, Australia; 4 Medical Education Development, School of Public Health and Community Medicine, UNSW Medicine, University of New South Wales, Sydney, Australia

**Keywords:** Collaborative learning, Small group learning, Group interaction

## Abstract

**Background:**

Collaboration is of increasing importance in medical education and medical practice. Students’ and tutors’ perceptions about small group learning are valuable to inform the development of strategies to promote group dynamics and collaborative learning. This study investigated medical students’ and tutors’ views on competencies and behaviours which promote effective learning and interaction in small group settings.

**Methods:**

This study was conducted at UNSW Australia. Five focus group discussions were conducted with first and second year medical students and eight small group tutors were interviewed. Data were transcribed verbatim and thematic analysis was conducted.

**Results:**

Students and tutors identified a range of behaviours that influenced collaborative learning. The main themes that emerged included: respectfulness; dominance, strong opinions and openness; constructiveness of feedback; active listening and contribution; goal orientation; acceptance of roles and responsibilities; engagement and enthusiasm; preparedness; self- awareness and positive personal attributes. An important finding was that some of these student behaviours were found to have a differential impact on group interaction compared with collaborative learning. This information could be used to promote higher quality learning in small groups.

**Conclusion:**

This study has identified medical students’ and tutors’ perceptions regarding interactional behaviours in small groups, as well as behaviours which lead to more effective learning in those settings. This information could be used to promote learning in small groups.

## Background

Small group, collaborative learning activities have become increasingly integral to higher education [1], and to medical education in particular [2]. This shift can be traced back to the inception of problem-based learning in the 1960s. The development of teamwork and collaborative skills has been acknowledged as fundamental for 21^st^ Century medical students and practitioners [3]. In order to promote collaborative skills in medical students and teamwork skills in doctors, regulatory bodies in medical education advocate a collaborative learning environment as opposed to one that is competitive [4, 5]. This has encouraged curricular reform in medical education, resulting in an increased use of collaborative, small group and active learning strategies, with reduced emphasis on both rote learning and passive procurement of knowledge [6–8].

Learning and working in small groups or teams brings together individuals with varied experiences, backgrounds, values and knowledge. Through discussion, interaction and negotiation, integration of the different perspectives results in shared understanding and problem solving [9]. According to Smith and MacGregor [10], “in most collaborative learning situations, students are working in groups of two or more, mutually searching for understanding, solutions, or meanings, or creating a product…..Questions, problems or the challenge to create something drive the group activity” (Pg. 11). This description highlights some important aspects of collaborative learning, which include communication between group members, sharing information, understanding each members roles and responsibility for working towards a common objective [1].

Despite many acknowledged advantages of small group collaborative learning, its effect on students’ learning remains controversial. Some studies report no difference in knowledge gained between small group learning and traditional teaching methods [6]. Furthermore, there have been reports that traditional lecture-based teaching has a greater impact on student knowledge acquisition than small group learning [11–13]. Understanding the factors which promote students’ development of understanding and acquisition of knowledge in small group learning is recognized as an important area that requires further research [14].

The majority of research in small group learning is centered on problem-based learning (PBL) environments. Meanwhile, a paucity of research exists regarding perceptions of effective collaborative learning in non-PBL curricula [15]. Several studies have investigated students’ perceptions of working and learning in small group, problem-based learning environments [16, 17]. Interactive behaviours in collaborative learning have also been researched extensively [18–22]. However, there is a lack of conclusive evidence on how the factors identified in research have an impact on the students’ learning in small groups [23]. Meanwhile, effective and ineffective behaviours in small group learning have also been explored [24, 25]. Despite the plethora of research in small group learning, there exists a gap in identifying the factors which are most influential in maximizing learning outcomes. The need for further research to explore the “essential elements in the learning environments and in students’ learning” has been highlighted [26]. According to Dolmans and Gijbels [26] the essential features of group learning relate not only to small group achievement but also to how students perceive and accept small group learning and in what way it encourages their learning.

Small group, collaborative learning can be viewed from the perspective of situated learning. Situated learning has a socio-cultural basis that views learning and development occurring in a dynamic interaction between the learner and the environment [2]. This view endorses an important principle that learning occurs through learners’ active participation, interaction and collaboration with others, for example in a small group learning context [2]. The collaborative learner transforms their understanding and develops their roles and responsibilities through participation and interaction with others in a group. Participation is one of the two important factors that is critical for learning [27].

The aim of this study was to explore, using qualitative methods, medical students’ and tutors’ perceptions of key collaborative behaviours which have an impact on small group, collaborative learning. The key behaviours that impact on collaborative learning have a positive impact on the quality of learning as well as on general group interaction [17]. The group discussions and interviews were designed to clarify and explore the differential impact of essential behaviours on these two important dimensions: the quality of learning and group interaction. Students’ and tutors’ perceptions provide useful information regarding group effectiveness [24, 25, 28] and this information can be used to inform, encourage and prepare future students for more effective collaborative learning.

## Methods

### Study setting

UNSW Medicine (the Faculty of Medicine at UNSW Australia) offers a 6-year undergraduate Medicine program. The program utilises an outcomes-based approach, guided by eight graduate capabilities which include outcomes relating to personal development and interactions (critical evaluation, reflection, communication and teamwork) as well as the canonical outcomes of medicine (understanding biomedical science, social and cultural aspects, patient assessment and management, and ethics) [29].

UNSW Medicine employs scenario-based learning (a variant of PBL) in the first and second year of the Medicine program [29]. Students spend 2 hours twice weekly, working on structured collaborative learning activities around a relevant clinical scenario. Each group comprises of 14–15 students with one facilitator/tutor. In the first session following the introduction of each scenario, students de-construct the scenario and identify relevant learning issues (supported by the facilitator). The subsequent sessions consist of a wide range of collaborative learning tasks designed to address all eight graduate capabilities.

In each teaching period (half a semester), summative assessment of students includes one individual assignment and one group project. The group project comprises three to four students working collaboratively to produce a written report and presentation. Students present their project progress during a scenario group session, at which time formative feedback is provided. The final project report is submitted for summative grading.

### Study design

First and second year medical students at UNSW were invited to participate in focus groups. These students were invited because of their current involvement in scenario group learning sessions and group projects. In addition, UNSW Medicine faculty members who are involved in facilitating small group learning activities were invited for a semi-structured interview. An invitation to participate in the study was sent by broadcast email.

### Focus group discussions with medical students

A total of 22 students participated in five focus group discussions, each of approximately 60–90 min in duration. Homogeneity in focus groups is important [30, 31], therefore separate sessions were conducted for first year and second year students. One focus group was conducted with year one students and four sessions were held with year two students. Each focus group had between three to seven participants, in keeping with recommended practice [15, 31, 32].

At the start of each group discussion, the moderator (MI) introduced herself, assured participants of the confidentiality of their responses and asked for permission to audio record the discussion. The moderator took notes of behaviours which emerged in the discussion. In the last five minutes of each focus group session, the collaborative behaviours identified by the participants were posted on a white board. Discussion was encouraged and students’ opinions were explored in terms of the nature of the impact of the identified behaviours regarding the effect of the behaviour on student learning and on the group interaction. In addition, this helped to optimize the accuracy of collected data via ‘participant verification’ [33] of the behaviours and their importance.

### Semi-structured interviews with small group tutors

The principal investigator (MI) conducted interviews with medical faculty members involved in scenario group facilitation with first year and second year medical students. A total of eight semi-structured interviews were conducted; each interview lasted between 30 to 45 min. The interview questions were designed using the “critical incident technique” [34]. The critical incident technique is widely used in medical education research and it focuses on understanding the most important professional experiences [35]. In this context the experiences of medical faculty on effective student behaviours in small group learning activities were explored. The interviewer asked open-ended questions about student behaviours that enhanced collaborative learning and behaviours which were ineffective; emerging issues were further explored.

Focus groups and interviews were conducted till the point of saturation was achieved which in this context was defined as when no new descriptions of behaviours or competencies emerged. In this study the final focus group discussion and the last interview conducted did not reveal any new behaviours or competencies in small group learning, which is in accordance with the definition of the concept of data saturation [36].

### Confidentiality and ethics

Participation in this study was voluntary and informed consent was obtained. During the time of data collection for this project, the moderator was not involved in scenario group facilitation or summative evaluation or grading of the student assignments or projects. This study received ethics approval from the Medical and Community Human Research Ethics Advisory Panel of the UNSW Australia (Reference Number: 2014-7-03).

### Data analysis

All focus group sessions and semi-structured interviews were audio recorded and transcribed verbatim. The data was analysed following Hesse-Biber and Leavy’s [37] four steps in data analysis. In the ‘data preparation’ phase the audio data was transcribed. Transcription is a valuable activity because listening to the data facilitates and starts the process of analysis and interpretation [37]. During the transcription, moderator and interviewer notes, including observations of non-verbal communication such as hand gestures and general agreement in the focus groups were added to the (transcribed) text. The two subsequent steps occurred simultaneously: data exploration and data reduction [37]. The transcripts were read, important phrases were highlighted and concepts were subsequently coded. The first author (MI) conducted the initial round of coding the data; the research team reviewed the transcripts and the coding process. Any discrepancies were discussed and resolved by the team. The coding process was performed keeping in view the questions that were asked during the focus group discussion and interviews. The emerging codes were further analysed and organised under common themes. In the final step of the analysis codes were further refined, organised into categories and emerging themes, and exemplar quotes identified.

## Results

**Fig. 1:**
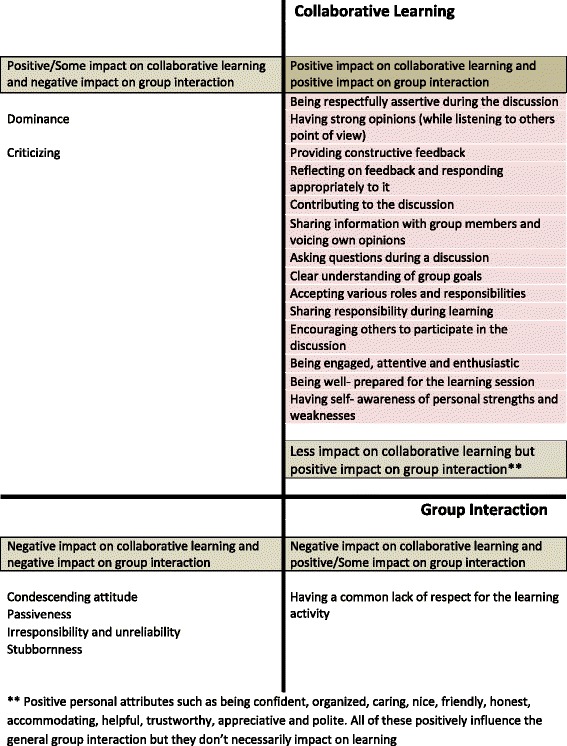
Impact of students’ behaviours on collaborative learning and group interaction

The important behaviours that emerged during focus group discussions and interviews can be categorised according to their impact on group interaction and collaborative learning (Fig. 1). The behaviours categorised in each quadrant in Fig. 1 are not organised in order of any reported effect. This differential impact of behaviours on learning and group interaction was clearly evident in both focus group discussions and tutor interviews. In particular, some behaviours improved general interaction between group members, but did not necessarily have a direct impact on learning, eg, positive personal attributes. Overall, fourteen behaviours emerged which had a positive impact on both group interactions and learning. These behaviours emerged as fundamental behaviours that impact on collaborative learning.

An interesting issue arose regarding dominant student behaviour and criticism. Both of these aspects were described as having a negative influence on group interaction, but also as potential triggers for learning within the group. Other behaviours were found to negatively influence both group interaction and learning, including: a condescending attitude; passivity; irresponsibility and unreliability; and stubbornness. Lastly, a common lack of respect for the learning activity was found to have a negative influence on learning, even though the group may interact well.

The findings from the focus group discussions and tutor interviews were analogous. The collaborative learning behaviours that were discussed in focus groups and interviews were very alike, and tutors and students emphasized similar behaviours that positively influenced the learning in small group settings. The themes which emerged from focus group discussion and interviews are therefore presented together in the order of how the discussion progressed about important behaviours in a collaborative learning environment.

### Theme: respectfulness

Tutors and students alike agreed that being respectful is a very important behaviour in both collaborative learning and group interaction. Several different aspects of respect were discussed, including: being respectful to peers during the discussion; respect for the learning activities; and respect for group work. However, being respectfully assertive during a discussion was stressed to be essential in learning as well as fostering a healthy group interaction, in majority of the focus group discussions. The following quotes illustrate this theme:

*“… because if you respect the other person you’re more likely to let them talk and also understand their perspective and still be able to keep and share your views.” (Focus group)*

*“You should treat the learning activities with respect…” (Focus group)*

*“I had a list of key things like respecting others and others’ views, not challenging each other in a negative way….. this also meant being punctual and coming in on time.” (R- Interview)*

A contrasting view on the concept of respect was that sometimes a common lack of respect for a learning activity managed to bring all students in agreement, but this had a very negative influence on learning. This observation highlights how important well designed learning activities are for effective group work.

*“……for some activities are a waste of everyone’s time, so we stop listening to our facilitator and once we realize this the drawback is that we all sit back and don’t want to work.” (Focus group)*

### Theme: dominance, strong opinions and openness

The concept of dominance stirred an interesting discussion among all focus groups. Dominant behaviour was discussed in terms of verbalising thoughts and ideas, as well as leading group learning. Students were of the view that dominance did lead people to contribute useful ideas. However, it often hampered others’ capacity to contribute to the discussion. The only area where first year and second year students differed was that first year students were more inclined to perceive that being active and dominant promoted small group discussion, whereas second year students emphasized that listening skills were important, in addition to strong opinions and dominant views. Meanwhile, tutors also agreed that dominance can start a discussion and thus provide a basis for learning within the group. Dominant students may negatively influence group interactions, but may in some instances helped students’ learning.

*“I think it depends on your personality…like some people thrive in that…umm kind of… dominant discussion. So they appreciate jumping in and feeding off ideas. Whereas other people think more of needing a space to think… and allow them to formulate ideas…” (Focus group)*

*“… for some of my peers, I think that they are a bit too dominative, like they are influential, so for some of my friends who are shy, it’s not that they don’t have ideas but they don’t have the opportunity to speak up in the group.” (Focus group)*

*“I guess the dominance of students can be a pro as well, in a way that it encourages discussion in students and gets the ball rolling if everyone is silent.” (R- Interview)*

A related concept was that of strong opinions and openness that were held by some students. This was described as similar to dominance in some ways, but with the added ability to listen to others’ points of view. Unlike dominance, strong opinion in students both positively influenced the learning and the group interaction.

*Student 1: “You want people to have some strong opinions which you can discuss… even if they don’t agree with yourself and it’s good to have that discussion.” (Focus group)*

*Student 2: “In my group project, the strong part is that everyone is giving ideas and discussing…… and we all kind of listen to each other and not just… like interrupt… we value others opinions.” (Focus group)*

### Theme: constructiveness of feedback

The constructive nature of feedback was highlighted. Giving and receiving feedback was considered an important ability that all students should develop. Students’ ability to reflect on the feedback and respond appropriately to it was emphasized. Constructive feedback helped students to both learn and establish a healthy group interaction. Some quotes to illustrate this are as follows:

*“I think being able to give feedback constructively… and not in terms of giving away the answer but gets the other person to think and learn and I think that is where this skill is very important.” (H- Interview)*

*“There should be feedback, but there must be a way in which you give it…like it makes it helpful or intimidating or overwhelming.” (Focus group)*

*“Students need to grasp the concept of constructive feedback earlier on and should be prepared to enact it.” (S- Interview)*

A more negative concept related to feedback was criticism. Criticism of their work and contributions to the group resulted in mixed feelings among students. Some opined that even though criticism can sometimes trigger learning, it does not help healthy group interactions. For example:

*“oh actually on criticism there is a difference…with our first presentation, I think we didn’t need to be too critical because it was just super uncomfortable and we really wanted to affirm each other…… we were ok with doing that and he just said that no this was a learning experience and that we need to recognize that this wasn’t good enough….. it was really strange and I dunno what to do… may be he was right…” (Focus group)*

### Theme: active listening and contribution

Verbal and non-verbal communication between group members was discussed in all focus groups and interviews. All participants agreed on the impact of discussion, contribution and participation on both group interaction and learning. Various aspects were highlighted as important for both group interaction and learning, including: the importance of active listening; encouraging others to participate in the discussion; sharing information; voicing opinions; and asking questions.

*“….For effective communication does not just involve speaking, but listening skills are especially important.” (Focus Group)*

*“I think actively contributing to ideas. There are some people who just sit there and like you to contribute ideas and all they say is I agree… I agree.” (Focus group)*

*“I mean obviously, a student who is asking questions, making comments and paying attention will learn better in a group.” (P- Interview)*

Students were of the view that failing to contribute to discussion by being passive during small group activity adversely affected others around them. This negatively influenced both learning and the general group interaction. For example, passive group response was de-motivating to the active students:

*“even if you do talk, you are received with silence…..I feel like you are being charged for contributing.” (Focus Group)*

Moreover the passiveness in the group was often due to quiet students who did not want to participate; this had a negative influence on the individual student and also the general group mood:

*“I dunno… I mean with the quieter students… it almost leads to being more subdued.” (Focus Group)*

### Theme: goal orientation

All focus groups agreed that orientation towards a set goal or outcome is important in both learning and group interaction. Students expected peers to have a clear understanding of the group goals, thus enabling the group to communicate and work together to achieve those goals.

Tutors were explicit in describing the goal orientation that was expected from students in collaborative learning:

*“I guess what I think of when students are collaborating that a group of students is working together towards a collective goal or understanding the task; with appreciating not only how to do something and why they are doing something and also the other skills that they need to deploy at the same time.” (S- Interview)*

### Theme: acceptance of roles and responsibilities

Faculty and students agreed on the value of accepting different roles and responsibilities during group interaction and learning. For example, leadership during the learning activity was emphasised in addition to the students’ roles and responsibilities to contribute to group work and discussion. Moreover, each student in a group had to share the responsibility for learning.

*“Everyone kind of hopes that someone will take the lead and step-up if necessary and take the lead- In the first instance no one wants to take the lead or be the leader….” (Focus Group)*

*“I found that our group goes off topic very easily so we need a person to remind us all that we were going off topic………..but having someone say, provided that they didn’t force it all the time…” (Focus Group)*

*“Yeah, more like leading by example rather than just forcing people to this….sort of that.. it was really good” (Focus Group)*

*“I guess adopting specific roles in the practice sessions…” (S-Interview)*

*“People who take responsibility for part of what you are doing and deliver it…” (M- interview)*

### Theme: engagement and enthusiasm

Tutors and students agreed that engagement in the learning activity, shown by attentiveness, enthusiasm, willingness to work, had a positive impact on both learning and group interaction:

*“Like being keen and being enthusiastic… this is really very helpful!” (Focus Group)*

In contrast, students identified lack of interest and contribution by group members as factors that result in ineffective group interaction and learning. Students highlighted disruptive behaviours, such as: ‘*showing lack of interest’; ‘Escaping or not taking responsibility’;* and ‘*Unable to be contacted when needed’.* Students did not feel comfortable in confronting group members who exhibit such behaviours. All groups agreed that disinterest by group members hindered discussion and created an uncomfortable atmosphere:

*“…..like if someone is floundering away and this not only holds them back but it also causes the whole group to hold back.” (Focus Group)*

*I think I experienced that…. They were uninterested like watching basketball while SG session was going on and it kind of made you not want to speak up as well because you would be like too keen or too enthusiastic and willing to learn when they are not willing to learn.” (Focus Group)*

### Theme: preparedness

An interesting theme which emerged was the level of preparation of group members. Participants expressed the view that adequate preparation allowed students to participate and communicate better in groups and thus helped in both group learning and interaction:

*“I think everyone should be prepared or at least be aware of where the scenario group is heading…” (Focus group)*

*“I always prefer to work with more active, conscientious and well prepared people in groups… what does everyone else think?” (Everyone agrees and says: ‘of-course,’ ‘most definitely’ and ‘yes’) (Focus Group)*

*“Students that are unprepared will most definitely not get a lot out of the teaching and the activities.” (P- Interview)*

### Theme: self-awareness

One aspect which was emphasized in several groups and interviews was being self- aware. This was discussed in the light of identifying personal strengths and weaknesses and the impact of this on contribution to the group work, discussion and communication:

*“I think that with self-awareness, you need to be assertive” (Focus group)*

*“… everyone has their strengths and flaws… so you need to accept it..”* and “*I know that I am good at this and I know I can help in this than I should be able to step up and say it that yea I have this skills and I am willing to contribute.” (Focus group)*

### Theme: positive personal attributes

Positive personal attributes were discussed in all focus groups. Students discussed behaviours that aided in developing collaboration between group members such as being organised, caring, pleasant, helpful, accommodating, polite and appreciative. Many students were of the view that these behaviours did not directly impact on learning. However, their impact on group interaction was important, and thus indirectly impacted on learning:

*“like being approachable and nice… even though that doesn’t guarantee the quality of work in the group.” (Focus group)*

*“I think being personable really helps….” (Focus group)*

## Discussion

The aim of this study was to explore medical students’ and tutors’ perceptions of collaborative behaviours which reported to have an impact on small group collaborative learning. An important finding of this study was the distinction between behaviours that impact on group interaction and those behaviours that impact on group learning. Positive group interaction by itself does not lead to effective learning, although it may often be a pre-requisite. Students and tutors clearly distinguished between behaviours that had a differential impact on learning and those that impacted on group interactions. Behaviours that positively influenced both interaction and learning could be regarded as fundamental factors in small group, collaborative learning.

The concept of respectful behaviour was highlighted as important and was described to have an impact on both learning and interaction in groups. Respect is a multifaceted concept because it means “different things to different people” ([38]; Pg. 707). The element of respect is imperative to collaboration because collaborative learning is described as “a partnership based on mutual respect for one another’s expertise, knowledge and skills” ([39]; Pg.14). This concept was discussed in many different contexts, such as respectful communication with colleagues and general respect for learning activities. Being respectfully assertive during an interaction enables the student to both listen to others’ points of view as well as discussing their own perspectives, which results in active involvement in group discussion and learning [40]. This active involvement has a positive impact on collaborative group learning.

The concept of dominance was well-debated in many focus groups and discussed in interviews. Dominant behaviour may well initiate a discussion but it can negatively influence group interactions. According to Hendry, Ryan [41], there can be several reasons for dominant student behaviour within a group, such as: natural predisposition; personal learning style; competitive strategy; and a need to gain respect. If dominant behaviour is a personal learning style preference, then that student might achieve better learning by being the dominant member. Also, dominance allows a person to contribute ideas that might trigger thinking among other members, which could indirectly lead to improved learning. Likewise, Balasooriya, di Corpo [42] have classified dominant students into two categories, based on the outcome of the dominance, ie “the dominant disruptive students” and “the highly enthusiastic dominant students” (Pg. 7). A related concept that emerged was strong opinions. This was described as a student who was assertive in discussing their views but at the same time was willing to listen to others’ points of view. Having strong opinions had a positive impact on both group interactions and collaborative learning.

Feedback has been recognized as a vital component of learning [43] and was highlighted during focus group discussions and interviews. The skill of giving and receiving constructive feedback was emphasized as important for medical students’ development; in addition to reflecting on the feedback received and responding appropriately to it. The other concept was that of criticism, which had a negative impact on group interactions, but could positively impact on individual students’ learning within the group.

The predominant theme that emerged from this study was the importance of active listening and contribution to the discussion. Students and tutors emphasized the importance of being willing and able to contribute, listen, discuss and to provide examples. This is consistent with previous studies regarding factors which enhance group productivity and learning [44].

In collaborative learning, members of the group work together as partners [10] and thus share a common goal or objective. In this study sharing a common goal had a positive impact on both learning and group interaction. Also, it was stressed in several group discussions and interviews that members need to accept different roles and responsibilities while working in the group. For example, the leadership role was widely discussed. The importance of leadership in teamwork and collaboration has been highlighted beyond health care [45]. In addition, it has been reported that allocation of specific roles to students in collaborative learning improves student efficiency, participation and satisfaction [46].

The level of student engagement and enthusiasm in group work has a positive influence on the quality of learning in such contexts [47]. Another important aspect which impacts on the learning and the interaction among group members is individual students’ preparation [48]. Measures to ensure student preparation have been incorporated in the design of team-based learning, which is a variant of collaborative learning in medicine [49].

An important goal and a pre-requisite of collaborative learning is the development of the self-regulated learner, which involves metacognitive skills such as planning, monitoring progress, and evaluating whether goals are achieved [50]. Individual students’ self-awareness in terms of understanding and reflecting on personal strengths and weaknesses influence their behaviour, participation and contribution in group learning.

The last theme of positive personal attributes was widely discussed among all focus groups and in interviews. The behaviours within this theme had a positive impact on group interaction, but both students and tutors agreed that they did not always impact on learning. Being personable is recommended for students participating in collaborative learning [51]. The findings of this study highlight that positive interaction by itself does not lead to higher quality learning. In addition, this study expands on the existing literature by identifying and distinguishing the factors that contribute to group interaction and their differential impact on collaborative learning.

### Limitations and avenues for future research

This study was conducted in the context of small group learning by junior medical students and tutors involved in small group facilitation. Therefore, our findings might not necessarily be generalisable to other contexts. It has been reported that the group practices of junior students remains stable over subsequent years [52]. The focus groups and interviews were conducted with volunteer students and faculty members, who consented to participate. We therefore cannot exclude the impact of sampling bias on our data, even though we conducted our focus groups and interviews until no new themes emerged.

Despite the limitations mentioned above, this study has identified student and tutor perceptions regarding fundamental collaborative behaviours which contribute to both group interactions and collaborative learning. Group dynamics and group interactions are recognised as important factors which promote student learning [53]. In that context, it is significant that a recent systematic review indicated that very few studies looked into the impact of group interactions on student learning in PBL setting and also the factors that influence on learning could not be clearly identified [23]. The findings of the present study could be employed to encourage, via feedback or student evaluations, essential behaviours for learning by individual students in small groups. This is in accordance with data showing that medical students perceive the need to promote collaborative capabilities through structured evaluation and feedback [54]. Data from the present study will be utilised to design an educational development instrument to promote critical collaborative behaviours in junior medical students. Future research could investigate the effect of promoting these behaviours on students’ learning.

Analogous to previous research, this study employed focus groups to collect student perceptions [15]. A rich discussion was generated among the participants in the focus groups [55], and students discussed and negotiated the collaborative behaviours of greatest importance. This discussion was supported, with students providing examples of situations and experiences of effective and ineffective collaborative behaviours. In addition, students were able to distinguish between the impact that behaviours had on group interaction and collaborative learning. The ‘critical incident technique’ was used to guide the semi-structure interview format with medical faculty involved in small group teaching and learning.

This study explored the perceptions of junior medical students and tutors about behaviours which contribute to small group interactions and collaborative learning. The findings highlight avenues for future research in collaborative learning, for example to compare and contrast these findings with perceptions of senior students and medical graduates. Such studies might provide an all-round perspective that could facilitate improved learning in collaborative settings.

## Conclusion

Our study participants (junior medical students and small group tutors) discussed the differential impact between behaviours which promote general group interaction and behaviours that impact on learning. The identified essential behaviours could provide the basis for developing educational interventions to promote these competencies in medical students. The findings of this study provide a perspective that could facilitate improved learning in collaborative settings.

## Abbreviations

PBL: Problem Based Learning
UNSW: University of New South Wales
